# National Hysteria

**Published:** 1899-03-04

**Authors:** 


### The Hospital

A Journal of

The Medical Sciences and Hospital Administration.

Vol. XXV.—No. 649.] Edited by Sir Henry Burdett, K.C.B., and Solomon C. Smith, M.D., M.R.C.P. [March 4, 1899.

### National Hysteria.

It is as true of communities as of persons that their histories are often written before they are born, and that the subtle influences of heredity sway masses of men no less than individuals. In the latter case, no doubt, the complication of events is often such as to defy analysis by the light of existing knowledge, and to render the apparent exceptions as little explicable as the manifest illustrations. However much this may be true, no physician would dispute that inheritance plays a prominent part in the causation of disease, or that this part is more conspicuous in the group of affections, commonly called "neuroses," than in any other; and we reasonably look forward to the progress of modern research, especially of research into the growth and descent of tissue, as being likely hereafter to render plain much that is still obscure, and to lift us from vague and general propositions to a level of absolute and communicable knowledge. In the meantime, it is hardly possible for the students of hereditary influences to be so engrossed by the observation of single instances as to neglect those which are displayed upon a larger scale; and of these it would be difficult to find a more conspicuous example than that which is at present being laid before us by the great nation from which we are divided by the English Channel, and with whose people we are united by so many ties of common interest; while, at the same time, our sympathies are so often alienated, and our consciences shocked, by aberrations of conduct or of opinion which it seems hardly possible to explain, and absolutely impossible not to condemn. France, at the present time, bears to other European states something of the relation which is borne by a hysterical girl to the more stable minded and better conducted members of her own family; and she not only offers a problem, or a series of problems, at once interesting and puzzling to all leading politicians whose aims and interests may well be endangered by her vagaries, but she also excites a totally different kind of curiosity in the breasts of less interested spectators who can scarcely determine whether her case is one for cajolery or for coercion, or as to the extent to which her conduct may be attributable, on the one hand to unreasoning impulse, on the other to sincere but mistaken conviction. Perhaps the solution of the doubt may be furnished by her history.

Until the middle or latter part of the reign of Louis XIV., France was at least as prosperous as any other part of western or central Europe. Her soil, no doubt, was often devastated by war, but her population was not excessive in relation to its productiveness, and therefore, as a rule, was fairly well nourished. The hideous misgovernment of the Grand Monarque and his successor, the wars which were carried on for many years, the oppression exercised by the nobles, and other causes of like kind, reduced the great bulk of the people to a state of semi-starvation, and caused every year the absolute death of multitudes from actual famine. The Revolution, which was mainly produced by these conditions, and in which they culminated, exposed the nervous systems of a half-starved people to the continuous strain of violent passions and of frenzied excitement; and the wars of Napoleon stripped the country of an enormous number of ablebodied men, leaving the next generation to be to a large extent the progeny of the feeble, or the useless. From the date of Waterloo to our own day there has been a constant succession of political and dynastic changes; and the generation now in full activity was either born during, or in childhood was dragged through, the terrible scenes of the war with Germany in 1870-71, with its destruction of all that a nation holds most dear, the slaughter of thousands of her bravest sons, the desecration of her soil, the utter humiliation of her pride, the associated evils of dire poverty and of prevailing pestilence, and the ceaseless suspicions and distrusts begotten of these evils, and leading to the "*nous sommes trahis*," which was too often accepted as an explanation of them. A very keen observer, the first Lord Lytton, remarked in 1869 that he saw no danger to the stability of the renewed Napoleonic dynasty, unless it were from the ficklenessof the French people. "They are so fickle," he continued, "that if they had had fifteen years of the Angel Gabriel, they would want to have a turn at Satan." It is impossible to consider their recent history, to remember the Fashoda incident, to make any attempt at sounding the depths of the miserable Dreyfus business, or to regard the witches' cauldron of petty and personal ambitions of which Paris is now the scene, without recurring to the definition of "irritability" as "debility excited," given years ago by Benjamin Travers, or without recognising how much there has been, in the incidents of the last two centuries, to produce at once the debility and the excitement. If France possessed statesmen in lieu of politicians, their chief ambition would surely be to give her such a period of repose as might allow the nervous systems of her children to recover from the excitements of the last two centuries, and to return to the normal standard of humanity. Unless this can be done she is likely to be a standing menace to the peace of Europe, to enter with a light heart into engagements beyond her strength, and to fail ignominiously in her endeavours to conduct them to successful issues.

